# ﻿Studies of *Diaporthe* (Diaporthaceae, Diaporthales) species associated with plant cankers in Beijing, China, with three new species described

**DOI:** 10.3897/mycokeys.98.104156

**Published:** 2023-05-29

**Authors:** Yukun Bai, Lu Lin, Meng Pan, Xinlei Fan

**Affiliations:** 1 The Key Laboratory for Silviculture and Conservation of Ministry of Education, Beijing Forestry University, 100083, Beijing, China Beijing Forestry University Beijing China

**Keywords:** Canker disease, Diaporthales, phylogeny, plant disease, taxonomy

## Abstract

The genus *Diaporthe* (Diaporthaceae, Diaporthales) comprises endophytes, pathogens and saprophytes, inhabiting a wide range of woody hosts and resulting in serious canker disease. To determine the diversity of *Diaporthe* species associated with canker disease of host plants in Beijing, China, a total of 35 representative strains were isolated from 18 host genera. Three novel species (*D.changpingensis*, *D.diospyrina* and *D.ulmina*) and four known species (*D.corylicola*, *D.donglingensis*, *D.eres* and *D.rostrata*) were identified, based on morphological comparison and phylogenetic analyses using partial ITS, *cal*, *his3*, *tef1-α* and *tub2* loci. These results provide an understanding of the taxonomy of *Diaporthe* species associated with canker diseases in Beijing, China.

## ﻿Introduction

*Diaporthe* (Diaporthales, Sordariomycetes) was established by Fuckel (1867) with *D.alnea* as the type species. Members of *Diaporthe* are distributed worldwide on the leaves, branches, fruits or seeds of broad hosts and often regarded as endophytes, pathogens and saprobes (Maharachchikumbura et al. 2015, 2016; Huang et al. 2021; Yang et al. 2021; Cao et al. 2022). Several species in *Diaporthe* have been reported as pathogens causing severe canker diseases on economically and ecologically important plants (e.g. *Castanea*, *Citrus*, *Juglans*, *Pyrus* and *Vaccinium*) (Udayanga et al. 2014; Fan et al. 2015; Guo et al. 2020; Hilário et al. 2020; Jiang et al. 2021a). Currently, more than 1190 species epithets of *Diaporthe* have been listed in Index Fungorum (www.indexfungorum.org; accessed on 23 Mar 2023).

The sexual morph of *Diaporthe* generally has immersed ascomata and erumpent pseudostroma with elongated perithecial necks. Asci are unitunicate and sessile producing hyaline ascospores (Udayanga et al. 2011). The asexual morph of *Diaporthe* can be identified by ostiolate conidiomata, cylindrical phialides and three types (alpha, beta and gamma) of conidia. All of the three types of conidia are aseptate and hyaline, but alpha conidia are fusiform, usually biguttulate; beta conidia are filiform, straight or more often hamate, lack guttules; gamma conidia are fusiform to subcylindrical, multiguttulate (Udayanga et al. 2011; Gomes et al. 2013).

In the past, species identification criteria in *Diaporthe* was largely based on host specificity and morphological features (Rehner and Uecker 1994; Santos et al. 2010; Dissanayake et al. 2020; Jiang et al. 2021b). However, many *Diaporthe* species have no obvious selectivity for hosts, for example, *D.eres* can infect more than 280 hosts (https://nt.ars-grin.gov/fungaldatabases; accessed on 23 Mar 2023). Additionally, although morphological characteristics were proved to be related to the DNA sequence of most *Diaporthe* species (Guo et al. 2020), many of them with similar morphology are still genetically distinct (Fan et al. 2018a; Jiang et al. 2021a, b). Therefore, it is unreliable for accurate identification when host specificity and morphological features were used alone (Udayanga et al. 2011, 2014; Gomes et al. 2013; Yang et al. 2018). Currently, molecular characteristics were proved to be relied on more heavily than morphology (Castlebury et al. 2003; Crous and Groenewald 2005; Udayanga et al. 2012). The taxonomy of *Diaporthe* species is resolved, based on polyphasic taxonomic concepts including multi-gene phylogenetic and morphological analyses (Udayanga et al. 2012; Fan et al. 2015; Guo et al. 2020; Gao et al. 2021; Jiang et al. 2021a). Five gene regions are used in phylogenetic analyses, including nuclear ribosomal internal transcribed spacer (ITS), calmodulin (*cal*), histone H3 (*his3*), translation elongation factor 1-α (*tef1-α*) and β-tubulin (*tub2*) (Dissanayake et al. 2020; Guo et al. 2020; Gao et al. 2021). The identification of *Diaporthe* species has significantly improved since the polyphasic taxonomic concept was applied, for example, 19 *Diaporthe* species were identified as pathogens associated with pear shoot canker, based on the five loci sequence data coupled with morphology (Guo et al. 2020). Additionally, some issues about species boundaries of the species complex in *Diaporthe* were also well resolved, such as the *D.eres* species complex being investigated and identified as a single species (Hilário et al. 2021; Norphanphoun et al. 2022).

Beijing is the capital city in China and is located in the northern part of the north China Plain. It has a temperate semi-humid monsoon climate, with more than 1,000 species of tree hosts (Ma et al. 1995; Liu et al. 2022). The pathogenic fungi of stem diseases in Beijing are diverse, especially *Diaporthe*. *Diaportheeres* have been identified from *CastaneaMollissima* and an additional five hosts (Yang et al. 2018); two *Diaporthe* species were commonly isolated from *Juglansmandshurica* (Zhu et al. 2019); *Diaporthedonglingensis*, *D.eres* and *D.huairouensis* were confirmed as pathogens of *Corylusheterophylla* (Bai et al. 2022). During the investigation of plant pathogens in Beijing, branches with typical canker symptoms were collected and subsequently identified combining modern taxonomic concepts. The present study aims to reveal the taxonomy and systematics of *Diaporthe* species with detailed descriptions of novel species.

## ﻿Materials and methods

### ﻿Collection, examination and isolation

Fresh specimens with typical ascomata/conidiomata were collected in the surveys of landscape plant canker in Beijing, China. Morphological features of the ascomata/conidiomata were determined by sectioning more than 30 fruiting bodies by hand vertically and horizontally under a stereomicroscope (M205 FA Leica). Over 50 asci/conidia were randomly selected to capture the micromorphological characteristics by using the compound microscope (DM2500 Leica) with differential interference contrast (DIC) optics. Isolates were obtained by cutting the mucoid asci/conidial mass with a sterile blade from the fruiting bodies to the surface of 1.8% potato dextrose agar (PDA) in a 9 cm Petri dish. Isolates were incubated at 25 °C until spores germinated. Hyphal tips were transferred to new PDA plates. The colour of the colony was assessed according to Rayner (1970). Axenic cultures were deposited in the China Forestry Culture Collection Centre (**CFCC**) and specimens were deposited in the Museum of Beijing Forestry University (**BJFC**).

### ﻿DNA extraction and PCR amplification

The cetyltrimethylammonium bromide (CTAB) method was used to extract the genomic DNA when enough mycelium of each isolate had grown on PDA for about five days (Doyle and Doyle 1990). PCR amplifications of five genes (ITS, *cal*, *his3*, *tef1-α* and *tub2*) were done by the primer pairs and PCR conditions listed in Table 1. The five partial loci have the same PCR mixtures including 10 μl Mix (Promega), 7 μl double deionised water, 1 μl of each primer and 1 μl template DNA. All of the amplified DNA were sequenced by the Qingke Biotechnology (Beijing, China). SeqMan v. 7.1.0 was used to check and assemble sequences for each of the gene sequences. The sequence data have been deposited in GenBank and their accession numbers have been listed in Table 2.

**Table 1.** Genes used in this study with PCR primers, primer DNA sequence, optimal annealing temperature.

Locus	PCR primers	PCR: thermal cycles: (Annealing temp. in bold)	Reference
ITS	ITS1/ITS4	(95 °C: 30 s, **48 °C**: 30 s, 72 °C: 1 min) × 35 cycles	White et al. (1990)
*cal*	CAL228F/CAL737R	(95 °C: 15 s, **54 °C**: 20 s, 72 °C: 1 min) × 35 cycles	Carbone and Kohn (1999)
*his3*	CYLH3F/H3-1b	(95 °C: 30 s, **57 °C**: 30 s, 72 °C: 1 min) × 35 cycles	Crous et al. (2004) Glass and Donaldson (1995)
*tef1-α*	EF1-728F/EF1-986R	(95 °C: 15 s, **54 °C**: 20 s, 72 °C: 1 min) × 35 cycles	Carbone and Kohn (1999)
*tub2*	T1(Bt2a)/Bt2b	(95 °C: 30 s, **55 °C**: 30 s, 72 °C: 1 min) × 35 cycles	Glass and Donaldson (1995) O’Donnell and Cigelnik (1997)

**Table 2.** Isolates of *Diaporthe* used in the molecular analyses in this study.

Species	Strain	Host	Origin	GenBank accession numbers				
				ITS	*cal*	*his3*	*tef1-α*	*tub2*
*Diaportheabsenteum*	LC 3924^T^	*Camelliasinensis*	China	KP267897	NA	KP293547	KP267971	KP293477
*Diaportheacaciigena*	CBS 129521^T^	*Acaciaretinodes*	Australia	KC343005	KC343247	KC343489	KC343731	KC343973
*Diaportheacericola*	MFLUCC 17-0956^T^	*Acernegundo*	Italy	KY964224	KY964137	NA	KY964180	KY964074
*Diaportheacerigena*	CFCC 52554^T^	*Acertataricum*	China	MH121489	MH121413	MH121449	MH121531	NA
	CFCC 52555	*Acertataricum*	China	MH121490	MH121414	MH121450	MH121532	NA
*Diaportheacerina*	CBS 137.27	*Acernegundo*	NA	KC343006	KC343248	KC343490	KC343732	KC343974
*Diaportheactinidiae*	ICMP 13683^T^	*Actinidiadeliciosa*	New Zealand	KC145886	NA	NA	KC145941	NA
*Diaportheacuta*	PSCG 047^T^	*Pyruspyrifolia*	China	MK626957	MK691125	MK726161	MK654802	MK691225
*Diaportheacutispora*	LC6161^T^	*Coffea* sp.	China	KX986764	KX999274	KX999235	KX999155	KX999195
*Diaporthealangii*	CFCC 52556^T^	*Alangiumkurzii*	China	MH121491	MH121415	MH121451	MH121533	MH121573
	CFCC 52557	*Alangiumkurzii*	China	MH121492	MH121416	MH121452	MH121534	MH121574
*Diaporthealbosinensis*	CFCC 53066	*Betulaalbosinensis*	China	MK432659	MK442979	MK443004	MK578133	MK578059
	CFCC 53067	*Betulaalbosinensis*	China	MK432660	MK442980	MK443005	MK578134	MK578060
*Diaporthealleghaniensis*	CBS 495.72^T^	*Betulaalleghaniensis*	Canada	MH121502	MH121426	MH121462	MH121544	MH121584
*Diaporthealnea*	CBS 146.46^T^	*Alnus* sp.	Netherlands	KC343008	KC343250	KC343492	KC343734	KC343976
*Diaportheamaranthophila*	MAFF 246900	*Amaranthustricolor*	Japan	LC459575	LC459583	LC459581	LC459577	LC459579
*Diaportheambigua*	CBS 114015	*Pyruscommunis*	South Africa	KC343010	KC343252	KC343494	KC343736	KC343978
*Diaportheampelina*	STE-U 2660	*Vitisvinifera*	France	NA	AY745026	NA	AY745056	NA
*Diaportheamygdali*	CBS 126679^T^	*Prunusdulcis*	Portugal	MH864208	KC343264	KC343506	KC343748	KC343990
*Diaportheanacardii*	CBS 720.97^T^	*Anacardiumoccidentale*	East Africa	KC343024	KC343266	KC343508	KC343750	KC343992
*Diaportheangelicae*	CBS 111592^T^	*Heracleumsphondylium*	Austria	KC343027	KC343269	KC343511	KC343753	KC343995
*Diaportheanhuiensis*	CNUCC 201901^T^	*Cunninghamialanceolata*	China	MN219718	MN224549	MN224556	MN224668	MN227008
*Diaportheapiculatum*	CFCC 53068	*Rhuschinensis*	China	MK432651	MK442973	MK442998	MK578127	MK578054
	CFCC 53069	*Rhuschinensis*	China	MK432652	MK44297	MK442999	MK578128	MK578055
*Diaportheaquatica*	IFRDCC 3051^T^	*Aquatichabitat*	China	JQ797437	NA	NA	NA	NA
*Diaporthearaucanorum*	CBS 145285^T^	*Araucariaaraucana*	Chile	MN509711	MN974277	NA	MN509733	MN509722
	CBS 145286	*Araucariaaraucana*	Chile	MN509712	NA	NA	MN509734	MN509723
*Diaporthearctii*	DP0482^T^	*Arctiumlappa*	Austria	KJ590736	KJ612133	KJ659218	KJ590776	KJ610891
*Diaporthearecae*	CBS 161.64^T^	*Arecacatechu*	India	KC343032	KC343274	KC343516	KC343758	KC344000
*Diaporthearengae*	CBS 114979^T^	*Arengaengleri*	Hong Kong	MF773664	KC343276	KC343518	KC343760	KC344002
*Diaporthearezzoensis*	MFLU 19-2883	*Cytisus* sp.	Italy	MT185503	NA	NA	NA	NA
*Diaportheaseana*	MFLUCC 12-0299a	Unknown	Thailand	KT459414	KT459464	NA	KT459448	KT459432
*Diaportheasheicola*	CBS 136967	*Vacciniumashei*	Chile	KJ160562	KJ160542	NA	KJ160594	KJ160518
*Diaportheaspalathi*	CBS 117169^T^	*Aspalathuslinearis*	South Africa	KC343036	KC343278	KC343520	KC343762	KC344004
*Diaportheaustralafricana*	CBS 111886^T^	*Vitisvinifera*	Australia	KC343038	KC343280	KC343522	KC343764	KC344006
*Diaportheaustraliana*	BRIP 66145^T^	*Macadamia* sp.	Australia	MN708222	NA	NA	MN696522	MN696530
*Diaporthebaccae*	CBS 136972^T^	*Vacciniumcorymbosum*	Italy	MK370623	MG281695	MF418264	KJ160597	MF418509
*Diaporthebatatas*	CBS 122.21^T^	*Ipomoeabatatas*	USA	KC343040	KC343282	KC343524	KC343766	KC344008
*Diaporthebauhiniae*	CFCC 53071	*Bauhiniapurpurea*	China	MK432648	MK442970	MK442995	MK578124	MK578051
*Diaporthebauhiniae*	CFCC 53072	*Bauhiniapurpurea*	China	MK432649	MK442971	MK442996	MK578125	MK578052
*Diaporthebauhiniae*	CFCC 53073	*Bauhiniapurpurea*	China	MK432650	MK442972	MK442997	MK578126	MK578053
*Diaporthebeilharziae*	BRIP 54792^T^	*Indigoferaaustralis*	Australia	JX862529	NA	NA	JX862535	KF170921
*Diaporthebenedicti*	SBen914	*Diaporthebenedicti*	USA	KM669929	KM669862	NA	KM669785	NA
*Diaporthebetulae*	CFCC 50469	*Betulaplatyphylla*	China	KT732950	KT732997	KT732999	KT733016	KT733020
	CFCC 50470	*Betulaplatyphylla*	China	KT732951	KT732998	KT733000	KT733017	KT733021
*Diaporthebetulicola*	CFCC 51128^T^	*Betulaalbosinensis*	China	KX024653	KX024659	KX024661	KX024655	KX024657
	CFCC 51129	*Betulaalbosinensis*	China	KX0246554	KX024660	KX024662	KX0246556	KX024658
*Diaporthebetulina*	CFCC 52560	*Betulaalbosinensis*	China	MH121495	MH121419	MH121455	MH121537	MH121577
	CFCC 52561	*Betulaalbosinensis*	China	MH121496	MH121420	MH121456	MH121538	MH121578
*Diaporthebicincta*	CBS 121004^T^	*Juglans* sp.	USA	KC343134	KC343376	KC343618	KC343860	KC344102
*Diaporthebiconispora*	ZJUD62	*Citrusmaxima*	China	KJ490597	NA	KJ490539	KJ490476	KJ490418
*Diaporthebiguttulata*	ZJUD47	*Citruslimon*	China	KJ490582	NA	KJ490524	KJ490461	KJ490403
*Diaporthebiguttusis*	CGMCC 3.17081	*Lithocarpusglabra*	China	KF576282	NA	NA	KF576257	KF576306
*Diaporthebohemiae*	CBS 143347^T^	*Vitisvinifera*	Czech Republic	MK300012	MG281710	MG281361	MG281536	MG281188
*Diaporthebrasiliensis*	CBS 133183^T^	*Aspidospermatomentosum*	Brazil	KC343042	KC343284	KC343526	KC343768	KC344010
*Diaporthecaatingaensis*	URM7485	*Tacingainamoena*	Brazil	KY085927	KY115598	NA	KY115604	KY115601
*Diaporthecamelliae-oleiferae*	HNZZ027^T^	*Camelliaoleifera*	China	MZ509555	MZ504685	MZ504696	MZ504707	MZ504718
*Diaporthecamelliae-sinensis*	SAUCC194.92	*Camelliasinensis*	China	MT822620	MT855699	MT855588	MT855932	MT855817
*Diaporthecamporesii*	JZB320143	*Urticadioidca*	Italy	MN533805	NA	NA	MN984254	MN561316
*Diaporthecamptothecicola*	CFCC 51632	*Camptothecaacuminata*	China	KY203726	KY228877	KY228881	KY228887	KY228893
*Diaporthecanthii*	CPC 19740	*Canthiuminerme*	South Africa	JX069864	NA	NA	NA	NA
*Diaporthecaryae*	CFCC 52563	*Caryaillinoinensis*	China	MH121498	MH121422	MH121458	MH121540	MH121580
	CFCC 52564	*Caryaillinoinensis*	China	MH121499	MH121423	MH121459	MH121541	MH121581
*Diaporthecassines*	CPC 21916	*Cassineperagua*	South Africa	KF777155	NA	NA	KF777244	NA
*Diaporthecaulivora*	CBS 127268	*Glycinemax*	Croatia	MH864501	KC343287	KC343529	KC343771	KC344013
*Diaporthecelastrina*	CBS 139.27^T^	*Celastrus* sp.	USA	KC343047	KC343289	KC343531	KC343773	KC344015
*Diaportheceleris*	CBS 143349^T^	*Vitisvinifera*	United Kingdom	MG281017	MG281712	MG281363	MG281538	MG281190
*Diaporthecercidis*	CFCC 52565^T^	*Cercischinensis*	China	MH121500	MH121424	MH121460	NA	MH121582
*Diaporthecercidis*	CFCC 52566	*Cercischinensis*	China	MH121501	MH121425	MH121461	NA	MH121583
*Diaporthechamaeropis*	CBS 454.81	*Chamaeropshumilis*	Greece	KC343048	KC343290	KC343532	KC343774	KC344016
***Diaporthechangpingensis***	**CFCC 58812^T^**	***Robiniapseudoacacia***	**China**	**OQ912925**	OQ910202	**OQ910234**	**OQ910264**	**OQ910292**
	**CFCC 58813**	***Robiniapseudoacacia***	**China**	**OQ912926**	**OQ910203**	**OQ910235**	**OQ910265**	**OQ910293**
*Diaporthecharlesworthii*	BRIP 54884m^T^	*Rapistrumrugostrum*	Australia	KJ197288	NA	NA	KJ197250	KJ197268
*Diaporthechensiensis*	CFCC 52567^T^	*Abieschensiensis*	China	MH121502	MH121426	MH121462	MH121544	MH121584
	CFCC 52568	*Abieschensiensis*	China	MH121503	MH121427	MH121463	MH121545	MH121585
*Diaporthechongqingensis*	PSCG 435^T^	*Pyruspyrifolia*	China	MK626916	MK691209	MK726257	MK654866	MK691321
*Diaporthechromolaenae*	MFLUCC 17-1422^T^	*Chromolaenaodorata*	Thailand	MT214456	NA	NA	NA	NA
*Diaporthecichorii*	MFLUCC 17-1023^T^	*Cichoriumintybus*	Italy	KY964220	KY964133	NA	KY964176	KY964104
*Diaporthecinnamomi*	CFCC 52569^T^	*Cinnamomum* sp.	China	MH121504	NA	MH121464	MH121546	MH121586
	CFCC 52570	*Cinnamomum* sp.	China	MH121505	NA	MH121465	MH121547	MH121587
*Diaporthecissampeli*	CPC 27302^T^	*Cissampeloscapensis*	South Africa	KX228273	NA	KX228366	NA	KX228384
*Diaporthecitri*	AR3405	*Citrus* sp.	USA	KC843311	KC843157	KJ420881	KC843071	KC843187
	CFCC 53079	*Citrussinensis*	China	MK573940	MK574579	MK574595	MK574615	MK574635
*Diaporthecitriasiana*	CGMCC 3.15224	*Citrusunshiu*	China	JQ954645	KC357491	KC490515	JQ954663	KC357459
*Diaporthecitrichinensis*	CGMCC 3.15225	*Citrus* sp.	China	JQ954648	KC357494	NA	JQ954666	NA
*Diaporthecollariana*	MFLU 17-2770^T^	*Magnoliachampaca*	Thailand	MG806115	MG783042	NA	MG783040	MG783041
*Diaporthecompactum*	LC3083^T^	*Camelliasinensis*	China	KP267854	NA	KP293508	KP267928	NA
*Diaportheconica*	CFCC 52571^T^	*Alangiumchinense*	China	MH121506	MH121428	MH121466	MH121548	MH121588
	CFCC 52572	*Alangiumchinense*	China	MH121507	MH121429	MH121467	MH121549	MH121589
*Diaportheconstrictospora*	GZCC 19-0065	Unknown	China	MT385947	MT424718	MW022487	MT424682	MT424702
	GZCC 19-0084^T^	Unknown	China	MT385948	MT424719	MW022487	MT424683	MT424703
*Diaportheconvolvuli*	CBS 124654^T^	*Convolvulusarvensis*	Turkey	KC343054	KC343296	KC343538	KC343780	KC344022
*Diaporthecoryli*	CFCC 53083^T^	*Corylusmandshurica*	China	MK432661	MK442981	MK443006	MK578135	MK578061
	CFCC 53084	*Corylusmandshurica*	China	MK432662	MK442982	MK443007	MK538176	MK578062
*Diaporthecorylicola*	CFCC 53986^T^	*Corylusheterophylla*	China	MW839880	MW836684	MW836717	MW815894	MW883977
	CFCC 54696	*Corylusheterophylla*	China	MW839867	MW836685	MW836718	MW815895	MW883978
	CFCC 54697	*Corylusheterophylla*	China	MW839882	MW836698	MW836731	MW815908	MW883991
***Diaporthecorylicola***	**CFCC 58824**	***Corylusheterophylla***	**China**	**OQ912927**	**OQ910203**	**NA**	**OQ910266**	**OQ910294**
	**CFCC 58825**	***Corylusheterophylla***	**China**	**OQ912928**	**OQ910204**	**NA**	**OQ910267**	**OQ910285**
*Diaporthecrataegi*	CBS 114435	*Crataegusrhipidophylla*	Sweden	KC343055	KC343297	KC343539	KC343781	KC344023
*Diaporthecrotalariae*	CBS 162.33^T^	*Crotalariaspectabilis*	USA	MH855395	JX197439	KC343540	GQ250307	KC344024
*Diaporthecrousii*	CAA 823	*Vacciniumcorymbosum*	Portugal	MK792311	MK883835	MK871450	MK828081	MK837932
*Diaporthecucurbitae*	DAOM 42078^T^	*Cucumis* sp.	Canada	KM453210	NA	KM453212	KM453211	KP118848
*Diaporthecuppatea*	CBS 117499^T^	*Aspalathuslinearis*	South Africa	MH863021	KC343299	KC343541	KC343783	KC344025
*Diaporthecynaroidis*	CBS 122676^T^	*Proteacynaroides*	South Africa	KC343058	KC343300	KC343542	KC343784	KC344026
*Diaporthecytosporella*	FAU461	*Citruslimon*	Italy	KC843307	KC843141	NA	KC843116	KC843221
*Diaporthedelonicis*	MFLU 16-1059	*Ipomoeabatatas*	China	KP990621	NA	KP990641	KP990651	KP990631
*Diaporthedestruens*	ZJUPD06	*Macadamia* sp.	South Africa	MN708229	NA	NA	MN696526	MN696537
*Diaporthediospyricola*	CPC 21169^T^	*Diospyroswhyteana*	South Africa	KF777209	NA	NA	NA	NA
*Diaporthediscoidispora*	ZJUD89	*Citrusunshiu*	China	KJ490624	NA	KJ490566	KJ490503	KJ490445
***Diaporthediospyrina***	**CFCC 58820^T^**	***Diospyroskaki***	**China**	**OQ912929**	**OQ910206**	**OQ910236**	**OQ910268**	**OQ910296**
	**CFCC 58821**	***Diospyroskaki***	**China**	**OQ912930**	**OQ910207**	**OQ910237**	**OQ910269**	**OQ910297**
*Diaporthedonglingensis*	CFCC 56581^T^	*Corylusheterophylla*	China	OM956090	NA	ON157951	ON157986	ON158021
	CFCC 57432	*Corylusheterophylla*	China	OM956091	NA	ON157952	ON157987	ON158022
***Diaporthedonglingensis***	**CFCC 58806**	***Corylusheterophylla***	**China**	**OQ912931**	**NA**	**OQ910238**	**OQ910270**	**OQ910298**
	**CFCC 58807**	***Corylusheterophylla***	**China**	**OQ912932**	**NA**	**OQ910239**	**OQ910271**	**OQ910299**
*Diaporthedorycnii*	MFLUCC 17-1015^T^	*Dorycniumhirsutum*	Italy	KY964215	NA	NA	KY964171	KY964099
*Diaporthedrenthii*	BRIP 66524^T^	*Macadamia* sp.	Australia	MN708229	NA	NA	MN696526	MN696537
*Diaportheelaeagni-glabrae*	LC4802	*Elaeagnusglabra*	China	KX986779	KX999281	KX999251	KX999171	KX999212
*Diaportheellipicola*	CGMCC 3.17084^T^	*Lithocarpusglaber*	China	KF576270	NA	NA	KF576245	KF576294
*Diaportheellipsospora*	GZCC 19-0231^T^	decaying woody	Guizhou, China	MT385949	MT424720	MW022488	MT424684	MT424704
*Diaportheendophytica*	CBS 133811^T^	*Schinusterebinthifolius*	Brazil	KC343065	KC343307	KC343549	KC343791	KC344033
*Diaportheeres*	AR5193^T^	*Ulmus* sp.	Germany	KJ210529	KJ434999	KJ420850	KJ210550	KJ420799
	CFCC 52575	*Castaneamollissima*	China	MH121510	NA	MH121470	MH121552	MH121592
	CFCC 52576	*Castaneamollissima*	China	MH121511	MH121432	MH121471	MH121553	MH121593
	CFCC 52577	*Acanthopanaxsenticosus*	China	MH121512	MH121433	MH121472	MH121554	MH121594
	CFCC 52578	*Sorbus* sp.	China	MH121513	MH121433	MH121473	MH121555	MH121595
*Diaportheeres*	CFCC 52579	*Juglansregia*	China	MH121514	NA	MH121474	MH121556	NA
	CFCC 52580	*Meliaazedarace*	China	MH121515	NA	MH121475	MH121557	MH121596
	CFCC 52581	*Rhododendrsimsii*	China	MH121516	NA	MH121476	MH121558	MH121597
***Diaportheeres***	**CFCC 58816**	***Corylusheterophylla***	**China**	**OQ912953**	**OQ910228**	**NA**	**OQ910288**	**OQ910320**
	**CFCC 58817**	***Corylusheterophylla***	**China**	**OQ912954**	**OQ910229**	**NA**	**OQ910289**	**OQ910321**
	**CFCC 58818**	***Populus* sp.**	**China**	**OQ912949**	**OQ910226**	**OQ910258**	**NA**	**OQ910318**
	**CFCC 58819**	***Populus* sp.**	**China**	OQ912950	**OQ910227**	**OQ910259**	**NA**	**OQ910319**
	**CFCC 58826**	***Spiraeasalicifolia***	**China**	**OQ912955**	**OQ910230**	**OQ910260**	**NA**	**OQ910322**
	**CFCC 58827**	***Spiraeasalicifolia***	**China**	**OQ912956**	**OQ910231**	**OQ910261**	**NA**	**OQ910323**
	**CFCC 58831**	***Ailanthusaltissima***	**China**	**OQ912933**	**OQ910208**	**OQ910240**	**OQ910272**	**OQ910300**
	**CFCC 58832**	***Ailanthusaltissima***	**China**	**OQ912934**	**OQ910209**	**OQ910241**	**OQ910273**	**OQ910301**
	**CFCC 58833**	***Koelreuteriapaniculata***	**China**	**OQ912935**	**OQ910210**	**OQ910242**	**OQ910274**	**OQ910302**
	**CFCC 58834**	***Forsythiasuspensa***	**China**	**OQ912936**	**OQ910211**	**OQ910243**	**OQ910275**	**OQ910303**
	**CFCC 58835**	***Acerpalmatum***	**China**	**OQ912937**	**OQ910212**	**OQ910244**	**OQ910276**	**OQ910304**
	**CFCC 58836**	***Syringaoblata***	**China**	**OQ912938**	**OQ910213**	**OQ910245**	**OQ910277**	**OQ910305**
	**CFCC 58837**	***Cotinuscoggygria***	**China**	**OQ912939**	**OQ910214**	**OQ910246**	**OQ910278**	**OQ910306**
	**CFCC 58838**	***Platycladusorientalis***	**China**	**OQ912940**	**OQ910215**	**OQ910247**	**OQ910279**	**OQ910307**
	**CFCC 58839**	***Populus* sp.**	**China**	**OQ912941**	**OQ910216**	**OQ910248**	**OQ910280**	**OQ910308**
	**CFCC 58840**	***Populus* sp.**	**China**	**OQ912942**	**OQ910217**	**OQ910249**	**OQ910281**	**OQ910309**
	**CFCC 58841**	***Pinusarmandii***	**China**	**OQ912943**	**OQ910218**	**OQ910250**	**OQ910282**	**OQ910310**
	**CFCC 58842**	***Pinusarmandii***	**China**	**OQ912944**	**OQ910219**	**OQ910251**	**OQ910283**	**OQ910311**
	**CFCC 58845**	***Juglansmandshurica***	**China**	**OQ912945**	**OQ910220**	**OQ910252**	**OQ910284**	**OQ910312**
	**CFCC 58846**	***Pterocaryastenoptera***	**China**	**OQ912946**	**OQ910221**	**OQ910253**	**OQ910285**	**OQ910313**
	**CFCC 58847**	***Prunussalicina***	**China**	**OQ912947**	**OQ910222**	**OQ910254**	**OQ910286**	**OQ910314**
	**CFCC 58848**	***Prunussalicina***	**China**	**OQ912948**	**OQ910223**	**OQ910255**	**OQ910287**	**OQ910315**
*Diaportheeucalyptorum*	CBS 132525^T^	*Eucalyptus* sp.	China	MH305525	NA	NA	NA	NA
*Diaporthefoeniculacea*	CBS 111553	*Foeniculumvulgare*	Spain	MH854926	KC343343	KC343585	KC343827	KC344069
*Diaporthefoikelawen*	CBS 145189	*Drimyswinteri*	Chile	MN509713	MN974278	NA	MN509735	MN509724
*Diaporthefraxini-angustifoliae*	BRIP 54781^T^	*Fraxinusangustifolia*	Australia	JX862528	KT459462	NA	JX862534	NA
*Diaporthefraxinicola*	CFCC 52582^T^	*Fraxinuschinensis*	China	MH121517	MH121435	NA	MH121560	NA
	CFCC 52583	*Fraxinuschinensis*	China	MH121518	MH121436	NA	MH121559	NA
*Diaporthefructicola*	MAFF 246408^T^	*Passifloraedulis*	Japan	LC342734	LC342738	LC342737	LC342735	LC342736
*Diaporthefukushii*	MAFF 625034	*Pyruspyrifolia*	Japan	NA	KJ435023	KJ420868	NA	KJ420819
*Diaporthefulvicolor*	PSCG 051^T^	*Pyruspyrifolia*	China	MK626859	MK691132	MK726163	MK654806	MK691236
*Diaporthefusicola*	CGMCC 3.17087	*Lithocarpusglabra*	China	KF576281	KF576233	NA	KF576256	KF576305
*Diaportheganjae*	CBS 180.91^T^	*Cannabissativa*	USA	KC343112	KC343354	KC343596	KC343838	KC344080
*Diaportheganzhouensis*	CFCC 53087	Unknown	China	MK432665	MK442985	MK443010	MK578139	MK578065
	CFCC 53088	Unknown	China	MK432666	MK442986	MK443011	MK578140	MK578066
*Diaporthegarethjonesii*	MFLUCC 12-0542a	Unknown	Thailand	KT459423	KT459470	NA	KT459457	KT459441
*Diaporthegoulteri*	BRIP 55657a^T^	*Helianthusannuus*	Australia	KJ197290	NA	NA	KJ197252	KJ197270
*Diaporthegrandiflori*	SAUCC194.84^T^	*Heterostemmagrandiflorum*	China	MT822612	MT855691	MT855580	MT855809	MT855924
*Diaportheguangxiensis*	JZB320087^T^	*Vitisvinifera*	China	MK335765	MK736720	NA	MK523560	MK500161
*Diaporthegulyae*	BRIP 54025^T^	*Helianthusannuus*	Australia	NA	NA	NA	JN645803	KJ197271
*Diaportheguttulata*	CGMCC 3.20100^T^	Unknown	China	MT385950	MW022470	MW022491	MT424685	MT424705
*Diaporthehelianthi*	CBS 592.81^T^	*Helianthusannuus*	Serbia	KC343115	KC343357	KC343599	KC343841	KC344083
*Diaporthehelicis*	AR5211^T^	*Hederahelix*	France	KJ210538	KJ435043	KJ420875	KJ210559	KJ420828
*Diaportheheliconiae*	SAUCC194.77^T^	*Heliconiametallica*	China	MT822605	MT855684	MT855573	MT855802	MT855917
*Diaportheheterophyllae*	CPC 26215	*Acaciaheterophylla*	France	MG600222	MG600218	MG600220	MG600224	MG600226
*Diaportheheterostemmatis*	SAUCC194.85^T^	*Heterostemmagrandiflorum*	China	MT822613	MT855692	MT855581	MT855810	MT855925
*Diaporthehickoriae*	CBS 145.26^T^	*Caryaglabra*	USA	KC343118	KC343360	NA	KC343844	KC344086
*Diaporthehispaniae*	CBS 143351^T^	*Vitisvinifera*	Spain	MG281123	MG281820	MG281471	MG281644	MG281296
*Diaporthehongkongensis*	CBS 115448^T^	*Dichroafebrifuga*	China	MK304388	KC343361	KC343603	KC343845	KC344087
*Diaporthehuairouensis*	CFCC 56808	*Corylusheterophylla*	China	ON188788	ON157945	ON157982	ON158016	ON158051
	CFCC 56809	*Corylusheterophylla*	China	OM956120	ON157946	ON157981	ON158015	ON158050
*Diaporthehubeiensis*	JZB320123^T^	*Vitisvinifera*	China	MK335809	MK500235	NA	MK523570	MK500148
*Diaportheincompleta*	LC6754	*Camelliasinensis*	China	KX986794	KX999289	KX999265	KX999186	KX999226
*Diaportheinconspicua*	CBS 133813^T^	*Maytenusilicifolia*	Brazil	NA	KC343365	KC343607	KC343849	KC344091
*Diaportheinfecunda*	CBS 133812^T^	*Schinusterebinthifolius*	Brazil	KC343126	KC343368	KC343610	KC343852	KC344094
*Diaportheirregularis*	CGMCC 3.20092^T^	Unknown	China	MT385951	MT424721	NA	MT424686	MT424706
*Diaportheisoberliniae*	CPC 22549	*Isoberliniaangolensis*	Zambia	KJ869190	NA	NA	NA	KJ869245
*Diaporthejuglandicola*	CFCC 51134^T^	*Juglansmandshurica*	China	KU985101	KX024616	KX024622	KX024628	KX024634
	CFCC 51135	*Juglansmandshurica*	China	KU985102	KX024617	KX024623	KX024629	KX024635
*Diaporthejuglandigena*	CFCC 52584	*Juglansregia*	China	MH121519	MH121437	MH121477	MH121561	MH121598
	CFCC 52585	*Juglansregia*	China	MH121520	MH121438	MH121478	MH121562	MH121599
*Diaporthekadsurae*	CFCC 52586^T^	*Kadsuralongipedunculata*	China	MH121521	MH121439	MH121479	MH121563	MH121600
	CFCC 52587	*Kadsuralongipedunculata*	China	MH121522	MH121440	MH121480	MH121564	MH121601
*Diaporthekochmanii*	BRIP 54033^T^	*Helianthusannuus*	Australia	NA	NA	NA	JN645809	NA
*Diaporthekongii*	BRIP 54031^T^	*Helianthusannuus*	Australia	NA	NA	NA	NA	KJ197272
*Diaporthekrabiensis*	MFLUCC 17-2481^T^	*Bruguiera* sp.	Unknown	MN047101	NA	NA	MN433215	MN431495
*Diaporthelenispora*	CGMCC 3.20101^T^	Unknown	China	MT385952	MW022472	MW022493	MT424687	MT424707
*Diaporthelitchicola*	BRIP 54900^T^	*Litchichinensis*	Australia	LC041036	NA	NA	JX862539	NA
*Diaporthelitchii*	SAUCC194.22^T^	*Litchichinensis*	China	MT822550	MT855635	MT855519	MT855747	MT855863
*Diaporthelithocarpus*	CGMCC 3.15175^T^	*Lithocarpusglabra*	China	KC135104	KF576235	NA	KC153095	KF576311
*Diaporthelongicicola*	CGMCC 3.17089^T^	*Lithocarpusglabra*	China	KF576267	NA	NA	KF576242	KF576291
*Diaporthelongicolla*	FAU599	*Glycinemax*	USA	KJ590728	KJ612124	KJ659188	KJ590767	KJ610883
*Diaporthelongispora*	CBS 194.36^T^	*Ribes* sp.	Canada	MH855769	KC343377	KC343619	KC343861	KC344103
*Diaporthelonicerae*	MFLUCC 17-0963^T^	*Lonicera* sp.	Italy	KY964190	KY964116	NA	KY964146	KY964073
*Diaporthelusitanicae*	CBS 123212^T^	*Foeniculumvulgare*	Portugal	MH863279	KC343378	KC343620	KC343862	KC344104
*Diaporthelutescens*	SAUCC194.36^T^	*Chrysalidocarpuslutescens*	China	MT822564	MT855647	MT855533	MT855761	MT855877
*Diaporthemacadamiae*	BRIP66526^T^	*Macadamia* sp.	Australia	MN708230	NA	NA	MN696528	MN696539
*Diaporthemachili*	SAUCC194.111^T^	*Machiluspingii*	China	MT822639	MT855718	MT855606	MT855951	MT855836
*Diaporthemacintoshii*	BRIP 55064a^T^	*Rapistrumrugosum*	Australia	KJ197289	NA	NA	KJ197251	KJ197269
*Diaporthemahothocarpus*	CGMCC 3.15181	*Lithocarpusglabra*	China	KC153096	NA	NA	KC153087	KF576312
*Diaporthemalorum*	CAA 734	*Malusdomestica*	Portugal	KY435638	KY435658	KY435648	KY435627	KY435668
*Diaporthemarina*	MFLU 17-2622	NA	Thailand	MN047102	NA	NA	NA	NA
*Diaporthemaritima*	DAOM 695742^T^	*Picearuben*	Canada	KU552025	NA	NA	KU552023	KU574615
*Diaporthemasirevicii*	BRIP 54256	*Glycinemax*	Australia	KJ197277	NA	NA	KJ197238	KJ197256
*Diaporthemayteni*	CBS 133185^T^	*Maytenusilicifolia*	Brazil	KC343139	KC343381	KC343623	KC343865	KC344107
*Diaporthemaytenicola*	CPC 21896^T^	*Maytenusacuminata*	South Africa	KF777157	NA	NA	NA	KF777250
*Diaporthemediterranea*	SAUCC194.111	*Machiluspingii*	China	MT822639	MT855718	MT855606	MT855836	MT855951
*Diaporthemelastomatis*	SAUCC194.55^T^	*Melastomamalabathricum*	China	MT822583	MT855664	MT855551	MT855780	MT855896
*Diaporthemelonis*	CBS 435.87	*Glycinesoja*	Indonesia	KC343141	KC343383	KC343625	KC343867	KC344109
*Diaporthemiddletonii*	BRIP 54884e^T^	*Rapistrumrugosum*	Australia	KJ197286	NA	NA	KJ197248	KJ197266
*Diaportheminima*	GZCC19-0066^T^	Unknown	China	MT385953	MT424722	MW022496	MT424688	MT424708
*Diaportheminusculata*	GZCC19-0215^T^	Unknown	China	MT385957	MW022475	MW022499	MT424692	MT424712
*Diaporthemiriciae*	BRIP 54736j^T^	*Helianthusannuus*	Australia	KJ197282	NA	NA	KJ197244	KJ197262
*Diaporthemomicola*	MFLUCC 16-0113	*Prunuspersica*	China	KU557563	NA	KU557611	KU557631	KU55758
*Diaporthemultigutullata*	CFCC 53095	*Citrusmaxima*	China	MK432645	MK442967	MK442992	MK578121	MK578048
	CFCC 53096	*Citrusmaxima*	China	MK432646	MK442968	MK442993	MK578122	MK578049
*Diaporthemusigena*	CBS 129519^T^	*Musa* sp.	Australia	KC343143	KC343385	KC343267	KC343869	KC344111
*Diaporthemyracrodruonis*	URM7972^T^	*Myracrodruonurundeuva*	Unknown	MK205289	MK205290	NA	MK213408	MK205291
*Diaportheneilliae*	CBS 144.27^T^	*Spiraea* sp.	USA	KC343144	KC343386	KC343628	KC343870	KC344112
*Diaportheneoarctii*	CBS 109490^T^	*Ambrosiatrifida*	USA	KC343145	KC343387	KC343629	KC343871	KC344113
*Diaportheneoraonikayaporum*	MFLUCC 14-1136	*Tectonagrandis*	Thailand	KU712449	KU749356	NA	KU749369	KU743988
*Diaporthenobilis*	CBS 587.79	*Pinusparviflora*	Japan	KC343153	KC343395	KC343637	KC343879	KC344121
*Diaporthenothofagi*	BRIP 54801^T^	*Nothofaguscunninghamii*	Australia	JX862530	NA	NA	JX862536	KF170922
*Diaporthenovem*	CBS 127269^T^	*Glycinemax*	Croatia	KC343155	KC343397	KC343639	KC343881	KC344123
*Diaportheocoteae*	CPC 26217^T^	*Ocoteabullata*	France	KX228293	NA	NA	NA	KX228388
*Diaportheoraccinii*	LC3166^T^	*Camelliasinensis*	China	KP267863	NA	KP293517	KP267937	KP293443
*Diaportheovalispora*	ZJUD93	*Citruslimon*	China	KJ490628	NA	KJ490570	KJ490507	KJ490449
*Diaportheovoicicola*	CGMCC 3.17093	*Lithocarpusglabra*	China	KF576265	KF576223	NA	KF576240	KF576289
*Diaportheoxe*	CBS 133186^T^	*Maytenusilicifolia*	Brazil	KC343164	KC343406	KC343648	KC343890	KC344132
*Diaporthepadina*	CFCC 52590^T^	*Padusracemosa*	China	MH121525	MH121443	MH121483	MH121567	MH121604
	CFCC 52591	*Padusracemosa*	China	MH121526	MH121444	MH121484	MH121568	MH121605
*Diaporthepandanicola*	MFLUCC 17-0607	Pandanaceae	Thailand	MG646974	NA	NA	NA	MG646930
*Diaportheparanensis*	CBS 133184^T^	*Maytenusilicifolia*	Brazil	KC343171	KC343413	KC343655	KC343897	KC344139
*Diaportheparapterocarpi*	CBS 137986	*Pterocarpusbrenanii*	Zambia	KJ869138	NA	NA	NA	KJ869248
*Diaportheparvae*	PSCG 035	*Pyrusbretschneideri*	China	MK626920	MK691169	MK726211	MK654859	MK691249
*Diaporthepascoei*	BRIP 54847^T^	*Perseaamericana*	Australia	MK111097	NA	NA	JX862538	KF170924
*Diaporthepassiflorae*	CPC 19183	*Passifloraedulis*	Netherlands	JX069860	NA	NA	NA	NA
*Diaporthepassifloricola*	CPC 27480^T^	*Passiflorafoetida*	Malaysia	KX228292	NA	KX228367	NA	KX228387
*Diaporthepenetriteum*	LC3215	*Camelliasinensis*	China	KP267879	NA	NA	KP293532	KP267953
*Diaportheperjuncta*	CBS 109745^T^	*Ulmusglabra*	Austria	KC343172	KC343414	KC343656	KC343898	KC344140
*Diaportheperseae*	CBS 151.73	*Perseagratissima*	Netherlands	KC343173	KC343415	NA	NA	NA
*Diaporthepescicola*	MFLUCC 16-0105	*Prunuspersica*	China	KU557555	KU557603	NA	KY400831	KU557579
*Diaporthephaseolorum*	AR4203^T^	*Phaseolusvulgaris*	USA	KJ590738	KJ612135	KJ659220	KJ590739	KJ610893
*Diaporthephillipsii*	CAA 817	*Vacciniumcorymbosum*	Portugal	MK792305	MK883831	MK871445	MK828076	MN000351
*Diaporthepimpinellae*	JZB320131^T^	*Pimpinellaperegrine*	Italy	MK874656	NA	MT373073	MT373074	MT373072
*Diaporthepodocarpi-macrophylli*	LC6155	*Podocarpusmacrophyllus*	Japan	KX986774	KX999278	KX999246	KX999167	KX999207
*Diaporthepometiae*	SAUCC194.72^T^	*Pometiapinnata*	China	MT822600	MT855679	MT855568	MT855797	MT855912
*Diaporthepseudoalnea*	CFCC 54190^T^	*Alnusglutinosa*	Netherlands	MZ727037	MZ753468	MZ781302	MZ816343	MZ753487
*Diaporthepseudomangiferae*	CBS 101339^T^	*Mangiferaindica*	Dominican Republic	KC343181	KC343423	KC343665	KC343907	KC344149
*Diaporthepseudophoenicicola*	CBS 176.77	*Mangiferaindica*	Iraq	KC343183	KC343425	KC343667	KC343909	KC344151
*Diaporthepseudotsugae*	MFLU 15-3228^T^	*Pseudotsugamenziesii*	Italy	KY964225	KY964138	NA	KY964181	KY964108
*Diaporthepsoraleae*	CPC 21634	*Psoraleapinnata*	South Africa	KF777158	NA	NA	KF777245	KF777251
*Diaporthepsoraleae-pinnatae*	CPC 21638^T^	*Psoraleapinnata*	South Africa	KF777159	NA	NA	NA	KF777252
*Diaporthepterocarpi*	MFLUCC 10-0571^T^	*Pterocarpusindicus*	Thailand	JQ619899	JX197451	NA	JX275416	JX275460
*Diaporthepterocarpicola*	MFLUCC 10-0580a^T^	*Pterocarpusindicus*	Thailand	JQ619887	JX197433	NA	JX275403	JX275441
*Diaporthepulla*	CBS 338.89^T^	*Hederahelix*	Yugoslavia	KC343152	KC343394	KC343636	KC343878	KC344120
*Diaporthepungensis*	SAUCC194.112^T^	*Elaeagnuspungens*	China	MT822640	MT855719	MT855607	MT855837	MT855952
*Diaporthepyracanthae*	CAA483	*Pyracanthacoccinea*	Portugal	KY435635	KY435645	KY435656	KY435625	KY435666
*Diaportheracemosae*	CPC 26646	*Euclearacemosa*	South Africa	MG600223	MG600219	MG600221	MG600225	MG600227
*Diaportheraonikayaporum*	CBS 133182	*Spondiasmombin*	Brazil	KC343188	KC343430	KC343672	KC343914	KC344156
*Diaportheravennica*	MFLUCC 16-0997	*Clematisvitalba*	Italy	NA	NA	NA	MT394670	NA
*Diaportherhusicola*	CPC 18191	*Rhuspendulina*	South Africa	JF951146	NA	NA	NA	NA
*Diaportherosae*	MFLUCC 17-2658	*Rosa* sp.	United Kingdom	MG828894	MG829273	NA	NA	MG843878
*Diaportherosicola*	MFLU 17-0646^T^	*Rosa* sp.	United Kingdom	MG828895	MG829274	NA	MG829270	MG843877
*Diaportherosiphthora*	COAD 2914^T^	*Rosa* sp.	Brazil	MT311197	MT313691	NA	MT313693	NA
*Diaportherossmaniae*	CAA 762^T^	*Vacciniumcorymbosum*	Portugal	MK792290	MK883822	MK871432	MK828063	MK837914
*Diaportherostrata*	CFCC 50062^T^	*Juglansmandshurica*	China	KP208847	KP208849	KP208851	KP208853	KP208855
	CFCC 50063	*Juglansmandshurica*	China	KP208848	KP208850	KP208852	KP208854	KP208856
***Diaportherostrata***	**CFCC 58843**	***Juglansmandshurica***	**China**	OQ912951	**NA**	**NA**	**NA**	**NA**
	**CFCC 58844**	***Juglansmandshurica***	**China**	**OQ912952**	**NA**	**NA**	**NA**	**NA**
*Diaportherudis*	AR3422^T^	*Laburnumanagyroides*	Austria	KC843331	KC843146	NA	KC843090	KC843177
*Diaporthesaccarata*	CBS 116311^T^	*Protearepens*	South Africa	KC343190	KC343432	KC343674	KC343916	KC344158
*Diaporthesackstonii*	BRIP 54669b^T^	*Helianthusannuus*	Australia	KJ197287	NA	NA	KJ197249	KJ197267
*Diaporthesalicicola*	BRIP 54825^T^	*Salixpurpurea*	Australia	JX862531	NA	NA	JX862537	KF170923
*Diaporthesambucusii*	CFCC 51986^T^	*Sambucuswilliamsii*	China	KY852495	KY852499	KY852503	KY852507	KY852511
	CFCC 51987	*Sambucuswilliamsii*	China	KY852496	KY852500	KY852504	KY852508	KY852512
*Diaportheschimae*	CFCC 53103	*Schimasuperba*	China	MK442640	MK442962	MK442987	MK578116	MK578043
	CFCC 53104	*Schimasuperba*	China	MK442641	MK442963	MK442988	MK578117	MK578044
	CFCC 53105	*Schimasuperba*	China	MK442642	MK442964	MK442989	MK578118	MK578045
*Diaportheschini*	CBS 133181^T^	*Schinusterebinthifolius*	Brazil	KC343191	KC343433	KC343675	KC343917	KC344159
*Diaportheschisandrae*	CFCC 51988^T^	*Schisandrachinensis*	China	KY852497	KY852501	KY852505	KY852509	KY852513
	CFCC 51989	*Schisandrachinensis*	China	KY852498	KY852502	KY852506	KY852510	KY852514
*Diaportheschoeni*	MFLU 15-1279^T^	*Schoenusnigricans*	Italy	KY964226	KY964139	NA	KY964182	KY964109
*Diaporthesclerotioides*	CBS 296.67	*Cucumissativus*	Netherlands	MH858974	KC343435	KC343677	KC343919	KC344161
*Diaporthesearlei*	BRIP 66528^T^	*Macadamia* sp.	Australia	MN708231	NA	NA	NA	MN696540
*Diaporthesennae*	CFCC 51636^T^	*Sennabicapsularis*	China	KY203724	KY228875	NA	KY228885	KY228891
	CFCC 51637	*Sennabicapsularis*	China	KY203725	KY228876	NA	KY228886	KY228892
*Diaporthesennicola*	CFCC 51634^T^	*Sennabicapsularis*	China	KY203722	KY228873	KY228879	KY228883	KY228889
	CFCC 51635	*Sennabicapsularis*	China	KY203723	KY228874	KY228880	KY228884	KY228890
*Diaportheserafiniae*	BRIP 55665a^T^	*Helianthusannuus*	Australia	KJ197274	NA	NA	KJ197236	KJ197254
*Diaportheshaanxiensis*	CFCC 53106	*Liana* sp.	China	MK432654	MK442976	MK443001	MK578130	NA
	CFCC 53107	*Liana* sp.	China	MK432655	MK432977	MK432002	MK578131	NA
*Diaporthesiamensis*	MFLUCC 10-0573a	*Dasymaschalon* sp.	Thailand	NA	JQ619897	NA	JX275393	JX275429
*Diaporthesilvicola*	CFCC 54191^T^	*Fraxinusexcelsior*	Netherlands	MZ727041	MZ753472	MZ753481	MZ816347	MZ753491
*Diaporthesojae*	FAU635^T^	*Glycinemax*	USA	KJ590719	KJ612116	KJ659208	KJ590762	KJ610875
*Diaporthespartinicola*	CPC 24951	*Spartiumjunceμm*	Spain	KR611879	NA	KR857696	NA	KR857695
*Diaporthespinosa*	PSCG 383^T^	*Pyruspyrifolia*	China	MK626849	MK691129	MK726156	MK654811	MK691234
*Diaporthesterilis*	CBS 136969^T^	*Vacciniumcorymbosum*	Italy	KJ160579	KJ160548	MF418350	KJ160611	KJ160528
*Diaporthestictica*	CBS 370.54	*Buxussampervirens*	Italy	KC343212	KC343454	KC343696	KC343938	KC344180
*Diaporthesubclavata*	ZJUD95	*Citrusunshiu*	China	KJ490630	NA	KJ490572	KJ490509	KJ490451
*Diaporthesubcylindrospora*	KUMCC 17-0151	Unknown	China	MG746629	NA	NA	MG746630	MG746631
*Diaporthesubellipicola*	KUMCC 17-0153	Unknown	China	MG746632	NA	NA	MG746633	MG746634
*Diaporthesubordinaria*	CBS 464.90	*Plantagolanceolata*	South Africa	KC343214	KC343456	KC343698	KC343940	KC344182
*Diaporthetaoicola*	MFLUCC 16-0117	*Prunuspersica*	China	KU557567	NA	NA	KU557636	KU557591
*Diaporthetarchonanthi*	CBS 146073^T^	*Tarchonanthuslittoralis*	South Africa	MT223794	NA	NA	MT223759	MT223733
*Diaporthetectonae*	MFLUCC 12-0777	*Tectonagrandis*	Thailand	KU712430	KU749345	NA	KU749359	KU743977
*Diaporthetectonendophytica*	MFLUCC 13-0471	*Tectonagrandis*	Thailand	KU712439	KU749354	NA	KU749367	KU743986
*Diaporthetectonigena*	MFLUCC 12-0767	*Camelliasinensis*	China	KX986782	KX999284	KX999254	KX999174	KX999214
*Diaportheterebinthifolii*	CBS 133180^T^	*Schinusterebinthifolius*	Brazil	KC343216	KC343458	KC343700	KC343942	KC344184
*Diaportheternstroemia*	CGMCC 3.15183	*Ternstroemiagymnanthera*	China	KC153098	NA	NA	KC153089	NA
*Diaporthethunbergii*	MFLUCC 10-0576a^T^	*Thunbergialaurifolia*	Thailand	JQ619893	JX197440	NA	JX275409	NA
*Diaporthethunbergiicola*	MFLUCC 12-0033^T^	*Thunbergialaurifolia*	Thailand	KP715097	NA	NA	KP715098	NA
*Diaporthetibetensis*	CFCC 51999^T^	*Juglandisregia*	China	MF279843	MF279888	MF279828	MF279858	MF279873
	CFCC 52000	*Juglandisregia*	China	MF279844	MF279889	MF279829	MF279859	MF279874
*Diaporthetorilicola*	MFLUCC 17-1051^T^	*Torilisarvensis*	Italy	KY964212	KY964127	NA	KY964168	KY964096
*Diaporthetoxica*	CBS 534.93^T^	*Lupinusangustifolius*	Australia	KC343220	KC343462	KC343704	KC343946	KC344188
*Diaporthetulliensis*	BRIP 62248a	*Theobromacacao*	Australia	KR936130	NA	NA	KR936133	KR936132
*Diaportheueckerae*	FAU656^T^	*Cucumismelo*	USA	KJ590726	KJ612122	KJ659215	KJ590747	KJ610881
*Diaportheukurunduensis*	CFCC 52592^T^	*Acerukurunduense*	China	MH121527	MH121445	MH121485	MH121569	NA
	CFCC 52593	*Acerukurunduense*	China	MH121528	MH121446	MH121486	MH121570	NA
***Diaportheulmina***	**CFCC 58828^T^**	***Ulmuspumil***a	**China**	**OQ912957**	**OQ910232**	**OQ910262**	**OQ910290**	**OQ910324**
	**CFCC 58829**	***Ulmuspumila***	**China**	**OQ912958**	**OQ910233**	**OQ910263**	**OQ910291**	**OQ910325**
	**CFCC 58830**	***Ulmuspumila***	**China**	**OQ912959**	**N**A	**NA**	**N**A	**NA**
*Diaportheundulata*	LC6624	Unknown	China	KX986798	NA	KX999269	KX999190	KX999230
*Diaportheunshiuensis*	CFCC 52594	*Caryaillinoensis*	China	MH121529	MH121447	MH121487	MH121571	MH121606
	CFCC 52595	*Caryaillinoensis*	China	MH121530	MH121448	MH121488	MH121572	MH121607
*Diaporthevaccinii*	CBS 160.32^T^	*Oxycoccusmacrocarpos*	USA	MH121502	MH121426	MH121462	MH121544	MH121584
*Diaporthevacuae*	CAA830	*Vacciniumcorymbosum*	Portugal	MK792306	MK883832	MK871446	MK828077	MK837928
*Diaporthevangueriae*	CBS 137985^T^	*Vangueriainfausta*	Zambia	KJ869137	NA	NA	NA	KJ869247
*Diaporthevawdreyi*	BRIP 57887a	*Psidiumguajava*	Australia	KR936126	NA	NA	KR936129	KR936128
*Diaporthevelutina*	LC4421	*Neolitsea* sp.	China	KX986790	NA	KX999261	KX999182	KX999223
*Diaportheverniciicola*	CFCC 53109	*Verniciamontana*	China	MK573944	MK574583	MK574599	MK574619	MK574639
	CFCC 53110	*Verniciamontana*	China	MK573945	MK574584	MK574600	MK574620	MK574640
*Diaportheviniferae*	JZB320071^T^	*Vitisvinifera*	China	MK341551	MK500119	NA	MK500107	MK500112
*Diaporthevirgiliae*	CMW 40748	*Virgiliaoroboides*	South Africa	KP247556	NA	NA	NA	KP247575
*Diaporthexishuangbanica*	LC6707	*Camelliasinensis*	China	KX986783	NA	KX999255	KX999175	KX999216
*Diaporthexunwuensis*	CFCC 53085	Unknown	China	MK432663	MK442983	MK443008	MK578137	MK578063
	CFCC 53086	Unknown	China	MK432664	MK442984	MK443009	MK578138	MK578064
*Diaportheyunnanensis*	LC6168	Unknown	China	KX986796	KX999290	KX999267	KX999188	KX999228
*Diaporthezaobaisu*	PSCG 031^T^	*Pyrusbretschneideri*	China	MK626922	NA	MK726207	MK654855	MK691245
*Diaporthellacorylina*	CBS 121124	*Corylus* sp.	NA	KC343004	KC343246	KC343488	KC343730	KC343972

**Note**: NA, not applicable. Strains in this study are marked in bold. Acronyms of culture collection: AR, DP, FAU isolates in culture collection of Systematic Mycology and Microbiology Laboratory, USDA-ARS, Beltsville, Maryland, USA; BRIP: Australian plant pathogen culture collection, Queensland, Australia; CAA: Personal Culture Collection Artur Alves, University of Aveiro, Aveiro, Portugal; CBS: Westerdijk Fungal Biodiversity Institute, Utrecht, The Netherlands; CFCC: China Forestry Culture Collection Center, China; CGMCC: China General Microbiological Culture Collection; CMW: culture collection (CMW) of the Forestry and Agricultural Biotechnology Institute; COAD: Coleção Octávio Almeida Drummond, Universidade Ferderal de Viçosa, Viçosa, Brazil; CPC: Collection Pedro Crous, housed at CBS; DAOM, Canadian Collection of Fungal Cultures or the National Mycological Herbarium, Plant Research Institute, Department of Agriculture (Mycology), Ottawa, Canada; IFRDCC: International Fungal Research and Development Centre Culture Collection, Chinese Academy of Forestry, Kunming, China; JZB, Culture collection of Institute of Plant and Environment Protection, Beijing Academy of Agriculture and Forestry Sciences, Beijing 100097, China. LC: working collection of Lei Cai, housed at Institute of Microbiology, CAS, China; MAFF: Ministry of Agriculture, Forestry and Fisheries, Tsukuba, Ibaraki, Japan; MFLUCC: Mae Fah Luang University Culture Collection; SAUCC: Shandong Agricultural University Culture Collection; ZJUD: Zhe Jiang University, China.

### ﻿Phylogenetic analyses

The sequences used in this study were aligned using MAFFT v. 6 (Katoh and Standley 2013) and corrected manually using MEGA v. 6.0 (Tamura et al. 2013). Reference sequences were obtained from the National Center for Biotechnology Information (NCBI), based on recent published literature associated with *Diaporthe* (Gao et al. 2021; Bai et al. 2022; Norphanphoun et al. 2022). The sequences of *Diaporthellacorylina* (CBS 121124) were included as outgroups in the polygenic *Diaporthe* analyses. The alignment, based on combined five concatenated sequences, were concatenated and aligned to compare with other species in *Diaporthe* to infer the phylogenetic position using Maximum Likelihood (ML) and Bayesian Inference (BI) analyses.

Maximum-likelihood (ML) analyses were conducted with 100 bootstrap support pseudoreplicates and the appropriate models for each gene using PhyML v. 3.0 (Guindon et al. 2010; Kozlov et al. 2019). Bayesian inference (BI) was conducted with a Markov Chain Monte Carlo (MCMC) algorithm in MrBayes v. 3.1.2 (Ronquist and Huelsenbeck 2003). MrModeltest v. 2.3 was used to estimate the best fit evolutionary models for each partitioned locus following the Akaike Information Criterion (AIC) (Posada and Crandall 1998). Two MCMC chains were run from random trees for 1,000,000 generations and stopped when the average standard deviation of split frequencies fell below 0.01. Trees were sampled every 100^th^ generation, resulting in a total of 10,000 trees. For each analysis, the first 25% of the trees were discarded as the burn-in phase and the remaining 75% trees were assessed to calculate the posterior probabilities (BPP) (Rannala and Yang 1996). Phylograms were viewed by using FigTree v. 1.3.1 and edited in Adobe Illustrator CS6 v. 16.0.0 (Rambaut and Drummond 2010).

## ﻿Results

### ﻿Phylogenetic analyses

**Figure 1.**
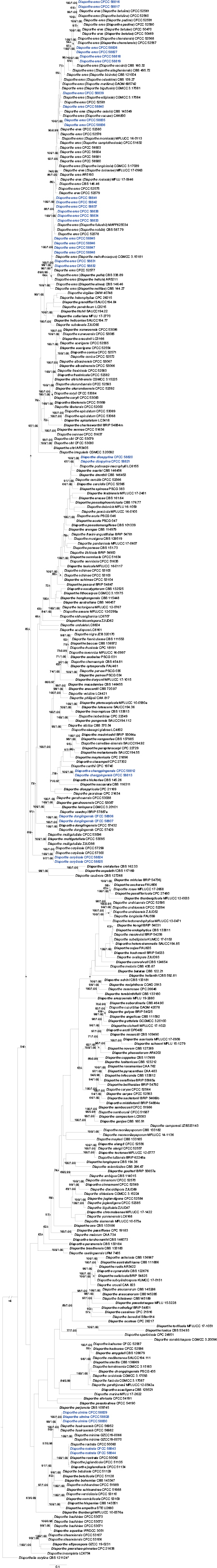
Phylogenetic tree of *Diaporthe* resulting from a Maximum-Likelihood (ML) analysis, based on the concatenated sequences from ITS, *cal*, *his3*, *tef1-α* and *tub2* genetic regions. Numbers above the branches indicate ML bootstraps (left, ML BS ≥ 50%) and Bayesian posterior probabilities (right, BPP ≥ 0.90). The tree is rooted with *Diaporthellacorylina*CBS 121124. Isolates from the present study are marked in blue and holotype isolates are indicated in bold.

The concatenated sequences of five genetic regions (ITS, *cal*, *his3*, *tef1-α* and *tub2*) were analysed to infer the interspecific relationships within *Diaporthe*. The dataset consisted of 343 sequences including the outgroup, *Diaporthellacorylina*CBS 121124. A total of 2,919 characters including gaps (547 for ITS, 578 for *cal*, 618 for *his3*, 619 for *tef1-α* and 557 for *tub2*) were included in the phylogenetic analysis. The topologies resulting from ML and BI analyses of the concatenated dataset were similar. ML bootstraps (ML BS ≥ 50%) and Bayesian posterior probabilities (BPP ≥ 0.95) have been shown above the branches (Fig. 1). In this study, 35 isolates formed seven clades representing seven species of *Diaporthe*, of which 22 isolates represented *D.eres*, CFCC 58824 and 58825 clustered together with *D.corylicola*, CFCC 58806 and 58807 grouped with *D.donglingensis* and CFCC 58843 and 58844 represented *D.rostrata*. The remaining seven isolates formed three distinct clades representing three new species which have been described below.

### ﻿Taxonomy

#### ﻿ Diaporthe changpingensis

Taxon classificationFungiDiaporthalesDiaporthaceae

Y.K. Bai & X.L. Fan sp. nov.

BE9423DE-27CA-5C5F-ABBE-72EC046B936E

847165

Fig. 2

##### Etymology.

Named after the place where it was first collected, Changping District, Beijing City.

##### Description.

Sexual morph not observed. Asexual morph: Conidiomata pycnidial, conical, immersed in bark, scattered, erumpent through the surface, with a solitary locule. Locule undivided, 620–830 μm diam. Conidiophores cylindrical, attenuate towards the apex, hyaline, phialidic, unbranched, slightly curved, 8.5–12.5 × 1.0–2.0 μm (av. = 10 ± 1.3 × 1.6 ± 0.3 μm, n = 50). Conidiogenous cells enteroblastic, phialidic, subcylindrical to cylindrical, 6.5–9.5 × 1.0–2.0 µm (av. = 8.0 ± 1.1 × 1.7 ± 0.2 µm, n = 50). Alpha conidia hyaline, aseptate, fusiform to oval, multi-guttulate, acute at both ends, 5.5–9.0 × 1.5–3.0 μm (av. = 6.5 ± 0.7 × 2.1 ± 0.5 μm, n = 50), L/W = 3.0–4.0 (av. = 3.5 ± 0.3, n = 50). Beta conidia hyaline, aseptate, filiform, straight or hamate, eguttulate, 13.0–19.0 × 1.0–2.0 μm (av. = 15.5 ± 1.5 × 1.5 ± 0.3 μm, n = 50), L/W = 9–11 (av. = 10 ± 0.4, n = 50).

**Figure 2.**
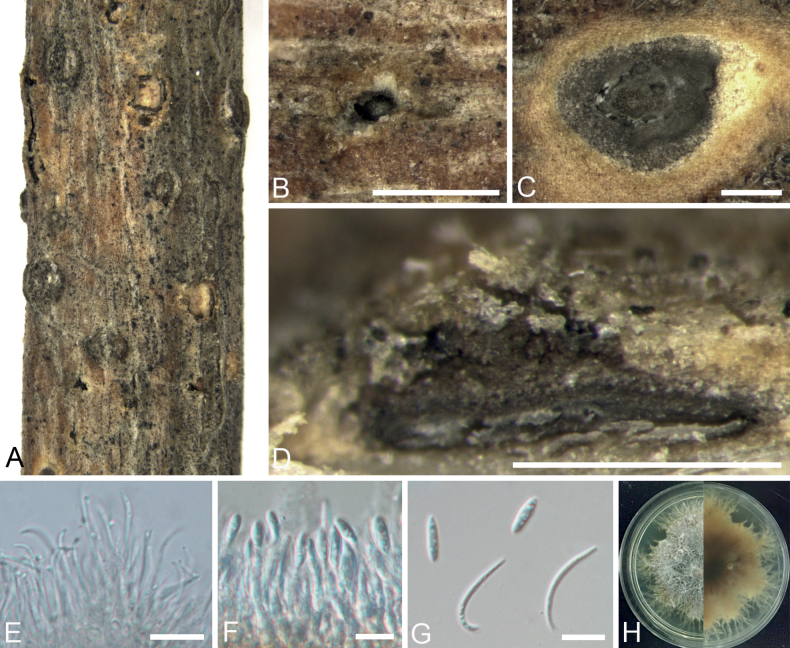
*Diaporthechangpingensis* from *Robiniapseudoacacia* (BJFC CF202212141) **A, B** habit of conidiomata on branch **C** transverse section of conidioma **D** longitudinal section through conidioma **E, F** conidiophores and conidiogenous cells **G** alpha and beta conidia **H** top (left) and bottom (right) sides of colonies on potato dextrose agar (PDA) after 7 days. Scale bars: 500 μm (**B, C**); 200 μm (**D**); 10 μm (**E–G**).

##### Culture characteristics.

Cultures on PDA initially white, growing slowly and entirely covering the 9 cm Petri dish after 14 days. The colonies flat, lacking aerial mycelium with an irregular edge. Conidiomata not observed on medium surface until 30 days.

##### Specimens examined.

China, Beijing City, Changping District, Baihujian Forest Park, 40°7'34.15"N, 116°5'30.26"E, on twigs and branches of *Robiniapseudoacacia*, 20 Aug 2022, Y.K. Bai, L. Lin & M. Pan (holotype BJFC CF202212141, ex-type living culture: CFCC 58812; other living culture: CFCC 58813).

##### Notes.

*Diaporthechangpingensis* was isolated from *Robiniapseudoacacia*. The molecular phylogenies of this species show a clearly different position in this study with high support (ML/BI = 100/1.00). This species appears most closely related to *D.canthii*. However, *D.changpingensis* can be distinguished from *D.canthii*, based on ITS, *tef1-α* and *tub2* loci (23/458 in ITS, 38/326 in *tef1-α* and 31/417 in *tub2*). Morphologically, *D.changpingensis* differs from *D.canthii* in having shorter alpha conidia (5.5–9.0 vs. 12.0–14.0 μm) and shorter beta conidia (13.0–19.0 vs. 18.0–25.0 μm) (Crous et al. 2012). Therefore, we described *D.changpingensis* as a novel species, based on morphology and sequence data.

#### ﻿ Diaporthe corylicola

Taxon classificationFungiDiaporthalesDiaporthaceae

H. Gao & X.L. Fan, Front. Cell. Infect. Microbiol. 11: 664366 (2021).

5BDAFD6B-9619-59F8-9D7E-A4274D072A0E

##### Description.

See Gao et al. (2021).

##### Specimens examined.

China, Beijing City, Yanqing District, Songshan National Nature Reserve, 40°30'4.32"N, 115°49'56.46"E, from branches of *Corylusheterophylla*, 17 Jun 2022, Y.K. Bai & X.L. Fan (BJFC CF202212148, cultures CFCC 58824 and 58825).

##### Notes.

*Diaporthecorylicola* was isolated from *Corylusheterophylla* in Beijing, China (Gao et al. 2021). This species is similar to *D.coryli* in culture morphology, but it can be distinguished by its longer and thinner alpha conidia (11.0–16.5 × 2.0–3.5 vs. 11.5–13.0 × 3.0–3.5 µm) (Gao et al. 2021). The isolates in this study clustered with *D.corylicola*, while the phylogram supported it belonging to this species because of the identical DNA sequence.

#### ﻿ Diaporthe diospyrina

Taxon classificationFungiDiaporthalesDiaporthaceae

Y.K. Bai & X.L. Fan sp. nov.

AFCF6F28-04FD-5CEB-90C7-BBBC070E20C3

847473

Fig. 3

##### Etymology.

Named after the host genus on which it was collected, *Diospyros*.

##### Description.

Sexual morph not observed. Asexual morph: Conidiomata pycnidial, conical, immersed in bark, scattered, erumpent through the surface, with a solitary locule. Locule undivided, 250–430 μm diam. Conidiophores cylindrical, attenuate towards the apex, hyaline, phialidic, unbranched, slightly curved, 10.0–27.0 × 0.5–2.0 μm (av. = 16.5 ± 4 × 1.3 ± 0.5 μm, n = 50). Conidiogenous cells enteroblastic, phialidic, subcylindrical to cylindrical, 4.5–8.0 × 1.0–2.0 µm (av. = 6.2 ± 1.2 × 1.3 ± 0.2 µm, n = 50). Alpha conidia hyaline, aseptate, oval, one guttulate at each end, 7.5–9.0 × 2.0–3.5 μm (av. = 8.2 ± 0.6 × 2.8 ± 0.3 μm, n = 50), L/W = 2.0–3.5 (av. = 2.7 ± 0.4, n = 50). Beta conidia not observed.

##### Culture characteristics.

Colonies with felty aerial mycelium initially white, growing to 80 mm after 3 days, with a uniform texture and regular edge, becoming umber after 9 days. Conidiomata black, distributed randomly at the marginal area.

##### Specimens examined.

China, Beijing City, Yanqing Distinct, Yeya Lake, 40°25'31.25"N, 115°51'36.34"E, from branches of *Diospyroskaki*, 14 Jun 2022, Y.K. Bai & X.L. Fan (holotype BJFC CF202212147, ex-type living culture: CFCC 58820; other living culture: CFCC 58821).

##### Notes.

*Diaporthediospyrina* and *D.diospyricola* were isolated from the same host genus *Diospyros* (Crous et al. 2013). Although *D.diospyricola* only has a sequence of the ITS locus, *D.diospyrina* can be distinguished from *D.diospyricola* by ITS (20/460). Morphologically, alpha conidia of *D.diospyrina* (7.5–9.0 μm) are longer than *D.diospyricola* (5.5–7.0 μm) (Crous et al. 2013). Therefore, the current two isolates (CFCC 58820 and 58821) were identified as a new species, *D.diospyrina*.

**Figure 3.**
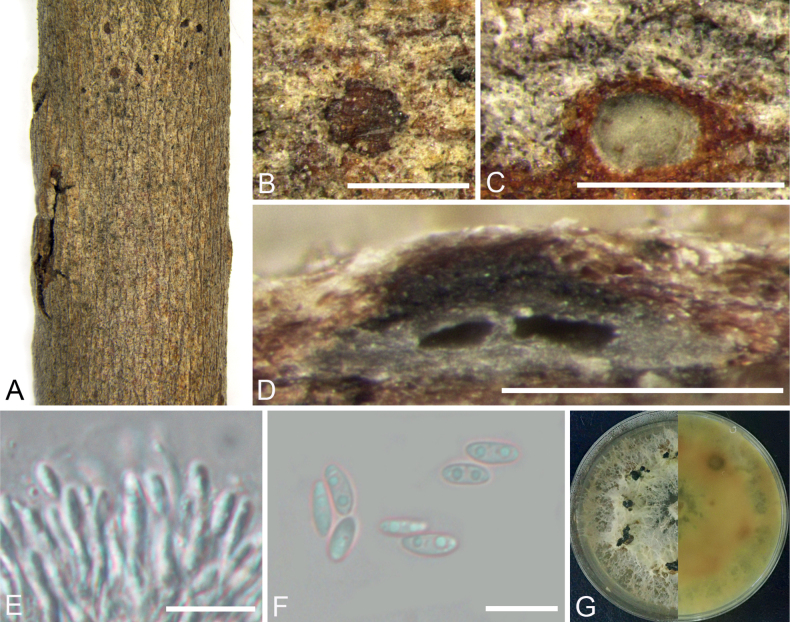
*Diaporthediospyrina* from *Diospyroskaki* (BJFC CF202212147) **A, B** habit of conidiomata on branch **C** transverse section of conidioma **D** longitudinal section through conidioma **E** conidiophores and conidiogenous cells **F** alpha conidia **G** top (left) and bottom (right) sides of colonies on potato dextrose agar (PDA) after 30 days. Scale bars: 500 μm (**B, C**); 250 μm (**D**); 10 μm (**E, F**).

#### ﻿ Diaporthe donglingensis

Taxon classificationFungiDiaporthalesDiaporthaceae

Y.K. Bai & X.L. Fan, Plant Pathol. 71: 1982 (2022).

868806F4-9754-59D8-9669-B7A0C8CB9393

##### Description.

See Bai et al. (2022).

##### Specimens examined.

China, Beijing City, Mentougou District, Mountain Dongling, Xiaolongmen Forestry Centre, 40°0'54.47"N, 115°29'36.24"E, on twigs and branches of *Corylusheterophylla*, 13 Jun 2022, Y.K. Bai & X.L. Fan (BJFC CF202212148, cultures CFCC 58806 and 58807).

##### Notes.

*Diaporthedonglingensis* was isolated from *Corylusheterophylla* in Beijing, China (Bai et al. 2022). Phylogenetically, isolates CFCC 58806 and 58807 clustered together with *D.donglingensis* with high statistical support (ML/BI = 100/1.00) (Fig. 1). Therefore, two isolates in this study were confirmed to be *D.donglingensis*.

#### ﻿ Diaporthe eres

Taxon classificationFungiDiaporthalesDiaporthaceae

Nitschke, Pyrenomyc. Germ. 2: 245 (1870).

E0B3719D-60D0-5FF0-AB89-4A55CDE79CA0

##### Remark.

Synonyms are listed in Hilário et al. (2021).

##### Description.

See Udayanga et al. (2014).

##### Specimens examined.

China, Beijing City, Mentougou District, Mountain Dongling, Xiaolongmen Forestry Centre, 39°59'59.42"N, 115°29'47.36"E, on twigs and branches of *Populus* sp., 15 Jun. 2022, Y.K. Bai & X.L. Fan (BJFC CF2022121411, cultures CFCC 58839 and 58840); Tongzhou District, 39°52'53.52"N, 116°43'45.35"E, on twigs and branches of *Acerpalmatum*, 11 Jun 2022, Y.K. Bai & X.L. Fan (BJFC CF2022121412, culture CFCC 58835); Tongzhou District, 39°52'53.25"N, 116°43'46.26"E, on twigs and branches of *Syringaoblata*, 11 Jun 2022, Y.K. Bai & X.L. Fan (BJFC CF2022121413, culture CFCC 58836); Tongzhou District, 39°52'53.28"N, 116°43'46.35"E, on twigs and branches of *Pinusarmandii*, 11 Jun 2022, Y.K. Bai & X.L. Fan (BJFC CF2022121414, cultures CFCC 58841 and 58842); Mentougou District, Mountain Dongling, Xiaolongmen Forestry Centre, 40°0'59.47"N, 115°29'47.34"E, on twigs and branches of *Cotinuscoggygria*, 15 Jun 2022, Y.K. Bai & X.L. Fan (BJFC CF2022121415, culture CFCC 58837); Mentougou District, Mountain Dongling, Xiaolongmen Forestry Centre, 40°0'59.28"N, 115°29'47.44"E, on twigs and branches of *Platycladusorientalis*, 15 Jun 2022, Y.K. Bai & X.L. Fan (BJFC CF2022121415, culture CFCC 58838); Mentougou District, Mountain Baihua, 39°59'54.38"N, 115°29'44.34"E, on twigs and branches of *Koelreuteriapaniculata*, 15 Jun 2022, Y.K. Bai & X.L. Fan (BJFC CF2022121416, culture CFCC 58833); Mentougou District, Mountain Baihua, 39°59'54.36"N, 115°29'44.35"E, on twigs and branches of *Forsythiasuspensa*, 26 Jun 2022, Y.K. Bai & X.L. Fan (BJFC CF2022121417, culture CFCC 58834); Mentougou District, Mountain Dongling, Xiaolongmen Forestry Centre, 39°59'59.31"N, 115°30'7.52"E, on twigs and branches of *Juglansmandshurica*, 15 Jun 2022, Y.K. Bai & X.L. Fan (BJFC CF2022121418, culture CFCC 58845); Mentougou District, Mountain Dongling, Xiaolongmen Forestry Centre, 40°0'59.36"N, 115°29'47.57"E, on twigs and branches of *Pterocaryastenoptera*, 15 Jun 2022, Y.K. Bai & X.L. Fan (BJFC CF2022121419, culture CFCC 58846); Fangshan District, Xiayunling National Forest Park, 39°44'35.32"N, 115°45'53.58"E, on twigs and branches of *Prunussalicina*, 23 Jun 2022, Y.K. Bai & X.L. Fan (BJFC CF2022121420, cultures CFCC 58847 and 58848); Mentougou District, Mountain Dongling, Xiaolongmen Forestry Centre, 39°59'59.36"N, 115°29'47.57"E, on twigs and branches of *Ailanthusaltissima*, 15 Jun 2022, Y.K. Bai & X.L. Fan (BJFC CF2022121421, cultures CFCC 58831 and 58832); Mentougou District, Beijing Songshan National Nature Reserve, 40°30'18.55"N, 115°50'34.24"E, from branches of *Corylusheterophylla*, 17 Jun 2022, Y.K. Bai & X.L. Fan (BJFC CF202212146, cultures CFCC 58816 and 58817); Daxing District, Gusang National Forest Park, 39°38'48.25"N, 116°33'25.44"E, from branches of *Populus* sp., 6 Jun 2021, X.L. Fan & L. Lin (BJFC CF202212143, cultures CFCC 58818 and 58819); Mentougou District, Mountain Dongling, Xiaolongmen Forestry Centre, 40°0'16.22"N, 115°29'33.65"E, from branches of *Spiraeasalicifolia*, 15 Jun 2022, Y.K. Bai & X.L. Fan (BJFC CF202212144, cultures CFCC 58826 and 58827).

##### Notes.

*Diaportheeres* was first described by Nitschke (1870) and isolated from *Ulmus* sp. in Germany. It is the most common species posing serious canker disease on diverse hosts (Gomes et al. 2013; Udayanga et al. 2014). In this study, 22 isolates were associated with canker diseases of 14 hosts genera including nine new host records in Beijing, China, which clustered in the *D.eres* species complex (Fig. 1). Therefore, these isolates were conformed to belong to *D.eres*, based on sequence data and morphology.

#### ﻿ Diaporthe rostrata

Taxon classificationFungiDiaporthalesDiaporthaceae

C.M. Tian, X.L. Fan & K.D. Hyde, Mycol. Prog. 14: 82 (2015).

307F1B52-2250-5A09-9423-1A37A1FC0E42

##### Remark.

Synonym is listed in Zhu et al. (2019).

##### Description.

See Fan et al. (2015).

##### Specimens examined.

China, Beijing City, Mentougou District, Mountain Dongling, Xiaolongmen Forestry Centre, 39°59'59.52"N, 115°29'47.26"E, on twigs and branches of *Juglansmandshurica*, 15 Jun 2022, Y.K. Bai & X.L. Fan (BJFC CF2022121410, cultures CFCC 58843 and 58844).

##### Notes.

*Diaportherostrata* was described as being associated with walnut dieback of *Juglansmandshurica* in China (Fan et al. 2015). The common symptom of this species was rostrate host tissue around the necks on infected branches (Fan et al. 2015). The current two isolates (CFCC 58843 and 58844) were identified as *D.rostrata* according to forming a fully supported clade with sequences from CFCC 50062, the ex-type of *D.rostrata* (ML/BI = 100/1.00).

#### ﻿ Diaporthe ulmina

Taxon classificationFungiDiaporthalesDiaporthaceae

Y.K. Bai & X.L. Fan sp. nov.

40CC76A2-5729-5B54-99C3-E088EB19CC3E

847184

Fig. 4

##### Etymology.

Named after the host genus on which it was collected, *Ulmus*.

##### Description.

Sexual morph: Ascostromata immersed in bark, erumpent, with 3–4 perithecial in black entostromata, conceptacle absent, 300–600 μm diam. Perithecia black, scattered, arranged circularly, ovoid to spherical, 250–380 μm (av. = 310 ± 30 μm, n = 30) diam. Asci 8-spored, unitunicate, clavate to cylindrical, sessile, 37–43 × 4.5–7 μm (av. = 40 ± 1.5 × 5.6 ± 0.5 μm, n = 50). Ascospores fusoid, hyaline, 2–4 guttulate, smooth-walled, 9–11 × 2–3.5 μm (av. = 9.9 ± 0.4 × 2.8 ± 0.4 μm, n = 50), L/W = 3–4 (av. = 3.4 ± 0.2, n = 50). Asexual morph not observed.

##### Culture characteristics.

Cultures with felty aerial mycelium are initially white, growing slowly and entirely covering the 9 cm Petri dish after 8 days, felty with a uniform texture and regular edge. Conidiomata were not observed until 30 days.

**Figure 4.**
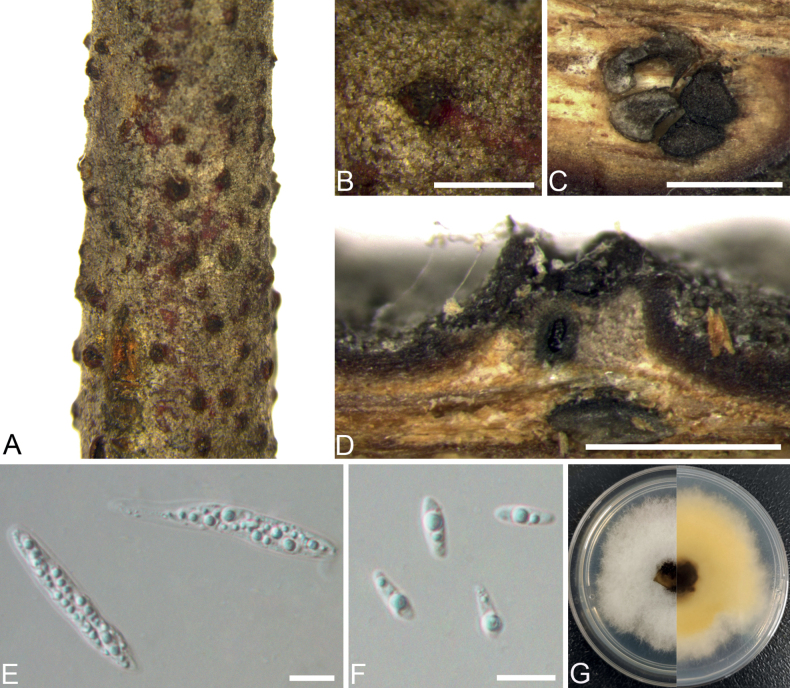
*Diaportheulmina* from *Ulmuspumila* (BJFC CF202212142) **A, B** habit of ascomata on branch **C** transverse section through ascomata **D** longitudinal section through ascomata **E** asci **F** ascospores **G** top (left) and bottom (right) sides of colonies on potato dextrose agar (PDA) after 7 days. Scale bars: 500 μm (**B–D**); 10 μm (**E, F**).

##### Specimens examined.

China, Beijing, Mentougou District, Mountain Dongling, Xiaolongmen Forestry Centre, 39°58'19.65"N, 113°12'39.24"E, from branches of *Ulmuspumila*, 16 Jun 2022, Y.K. Bai & X.L. Fan (holotype BJFC CF202212142, ex-type living culture: CFCC 58828; other living culture: CFCC 58829; *ibid*. BJFC CF2022121423, culture CFCC 58830).

##### Notes.

*Diaportheulmina* is associated with canker disease of *Ulmuspumila*. In this study, the isolates CFCC 58828 and 58829 formed a single-lineage clade with high support values (ML/BI = 100/1.00) and it appears to be most closely related to *D.huairouensis* (Fig. 1). *Diaportheulmina* differs from *D.huairouensis* isolated from *Corylusheterophylla* by host association (Bai et al. 2022). Phylogenetically, *D.ulmina* can be distinguished from *D.huairouensis* by base differences as follows: 16/466 for ITS, 4/420 for *cal*, 17/473 for *his3*, 34/329 for *tef1-α* and 10/420 for *tub2* (Bai et al. 2022). Therefore, *D.ulmina* is described as a new species.

## ﻿Discussion

The current study described three new species (*D.changpingensis*, *D.diospyrina* and *D.ulmina*) and four known species (*D.corylicola*, *D.donglingensis*, *D.eres* and *D.rostrata*), based on 35 isolates of *Diaporthe* in Beijing, China. The results indicate that *Diaporthe* species in Beijing are diverse and logical disease control strategies are required.

Since modern taxonomy approaches were applied, more than 40 novel species have been introduced in the recent five years (Fan et al. 2018b; Yang et al. 2018; Dissanayake et al. 2020; Guo et al. 2020; Hilário et al. 2020; Gao et al. 2021; Huang et al. 2021; Jiang et al. 2021b; Bai et al. 2022; Cao et al. 2022). Warmer climate and extensive application of chemicals in fungicides may lead to emergence of new species that are more resistant in northern China (Piao et al. 2010; Úrbez-Torres 2011; Manawasinghe et al. 2018; Jiang et al. 2022a, b). *Diaporthe* species pose a significant challenge to disease control due to their high species diversity and outstanding environmental adaptation.

Taxonomic identification of the *Diaporthe* species complexes is challenging. Norphanphoun et al. (2022) introduced 13 species complexes (*D.alnea*, *D.arecae*, *D.biconispora*, *D.carpini*, *D.decedens*, *D.oncostoma*, *D.pustulata*, *D.rudis*, *D.scobina*, *D.sojae*, *D.toxica*, *D.varians* and *D.vawdreyi*) to make the identification of *Diaporthe* species easier. The current phylogenetic analysis revealed that *D.donglingensis* clustered between the *D.decedens* and *D.oncostoma* complexes and the remaining isolates clustered in the *D.alnea*, *D.arecae*, *D.carpini*, *D.decedens* and *D.oncostoma* complexes (Fig. 1), of which the *D.alnea* complex was controversial. *Diaportheeres* was extensively studied and described as a complex by Udayanga et al. (2014). Fan et al. (2018b) treated four species (*D.biguttusis*, *D.ellipicola*, *D.longicolla* and *D.mahothocarpus*) as synonyms of the *D.eres* complex using a three genes matrix (*cal*, *tef1-α* and *tub2*). Then Hilário et al. (2021) treated 31 species in the *D.eres* complex as one species, based on the GCPSR principle and the coalescent-based species model (PTP). Currently, *D.eres* is included in the *D.alnea* complex by Norphanphoun et al. (2022). *Diaporthealnea* was used to describe it because *D.alnea* was the oldest name that was introduced in 1867 (Fuckel 1867). However, most of the species in this complex have been treated as synonyms of *D.eres* and *D.eres* was used most often in the past (Hilário et al. 2021). Therefore, we suggest using *D.eres* to describe this complex to make communication easier. In this study, we considered *D.eres* as a single species following Hilário et al. (2021). The largest isolation rate of *D.eres* (62.86%) revealed this species to be the most prevalent species in Beijing, which is consistent with Bai et al. (2015). As an important pathogen, it has a wide range of hosts, especially hosts in Rosaceae (https://nt.ars-grin.gov/fungaldatabases; accessed on 23 Mar 2023). In this study, *D.eres* were reported on 14 hosts including nine new hosts (*Ailanthusaltissima*, *Cotinuscoggygria*, *Forsythiasuspensa*, *Koelreuteriapaniculata*, *Pinusarmandii*, *Platycladusorientalis*, *Prunussalicina*, *Pterocaryastenoptera* and *Syringaoblata*). The pathogenicity of *D.eres* on these hosts should be evaluated in further studies.

Hazelnuts and walnuts are important plants for ecological forestation and economy and are suffering from various fungal pathogens. Over 40 species of fungi occurring on *Corylus* have been recorded in the Fungal database (https://nt.ars-grin.gov/fungaldatabases; accessed on 23 Mar 2023). *Diaportheeres* is the main cause of hazelnut defects in the Caucasus Region (Battilani et al. 2018). In this study, we accepted three species (*D.corylicola*, *D.donglingensis* and *D.eres*) inhabiting hazelnuts, of which *D.corylicola* was reported as the main species isolated form *Corylus* in Beijing (Gao et al. 2021). The comparisons show that the occurrence of *Diaporthe* species may associate with geographical and environmental factors. The distribution of *Diaporthe* species requires further studies. In terms of walnut, three species (*D.eres*, *D.rostrata* and *D.tibetensis*) have been reported causing canker disease in *Juglans* in China (Fan et al. 2015, 2018b). However, this study accepted *D.eres* and *D.rostrata* inhabiting *Juglans* in the present study in Beijing. These results proved that *Corylus* and *Juglans* could be infected by diverse species of *Diaporthe*. These fungi have become one of the main threats to hosts and pose serious environmental burdens. Therefore, preventative measures are required to control the diseases caused by *Diaporthe* species.

Most *Diaporthe* species occur on a wide host range, especially *D.eres* (Gomes et al. 2013). However, some of the species seem to be limited to a single host species in the current study. For example, *D.rostrata* is associated with canker diseases of *Juglansmandshurica*, which is consistent with the results of Zhu et al. (2019). Therefore, extensive sampling should be constructed in the future to better understand the host association of *Diaporthe* species.

## Supplementary Material

XML Treatment for Diaporthe changpingensis

XML Treatment for Diaporthe corylicola

XML Treatment for Diaporthe diospyrina

XML Treatment for Diaporthe donglingensis

XML Treatment for Diaporthe eres

XML Treatment for Diaporthe rostrata

XML Treatment for Diaporthe ulmina

## ﻿Additional information

### Author ORCIDs

Yukun Bai https://orcid.org/0000-0003-4433-2931

Meng Pan https://orcid.org/0000-0002-4580-0496

Xinlei Fan https://orcid.org/0000-0002-4946-4442

### Data availability

The data that support the findings of this study are available from the corresponding author upon reasonable request.
